# Biofilm formation, antimicrobial susceptibility, serogroups and virulence genes of uropathogenic *E. coli* isolated from clinical samples in Iran

**DOI:** 10.1186/s13756-016-0109-4

**Published:** 2016-04-01

**Authors:** Elahe Tajbakhsh, Parvin Ahmadi, Elham Abedpour-Dehkordi, Nazila Arbab-Soleimani, Faham Khamesipour

**Affiliations:** Department of Microbiology, Faculty of Basic Sciences, Shahrekord Branch, Islamic Azad University, Shahrekord, Iran; Faculty of Basic Sciences, Shahrekord Branch, Islamic Azad University, Shahrekord, Iran; Department of Microbiology, Damghan Branch, Islamic Azad University, Damghan, Iran; Cellular and Molecular Research Center, Sabzevar University of Medical Sciences, Sabzevar, Iran

**Keywords:** Antibiotic resistance pattern, Biofilm, Virulence genes, Uropathogenic *E. coli*, Urinary Tract Infection

## Abstract

**Background:**

Uropathogenic *Escherichia coli* O- Serogroups with their virulence factors are the most prevalent causes of UTIs. The present research performed to track common uropathogenic *E.coli* serogroups, antibiotic resistance pattern of strains and prevalence of virulence genes in isolations having the ability to constitute biofilm.

**Methods:**

In this research 130 *E.coli* isolation from patients having UTI symptoms were collected and antimicrobial resistance pattern was performed by Kirby-Bauer method. Polymerase chain reaction was done using primer pairs to identify common serogroups of uropathogenic *E.coli* and studying virulence genes in isolations creating biofilm.

**Results:**

Among 130 *E.coli* isolates, 80 (61.53 %) were able to make biofilm that 15 isolates (18.75 %) indicated strong reaction, 20 (25 %) of medium and 45 (56.25 %) of weak biofilm reaction. Among isolations creating biofilm, the highest resistance reported to Ampicillin (87.5 %) and the lowest to Nitrofurantoin (3.75 %). The frequency of *fimH*, *pap*, *sfa* and *afa* genes in isolations having the ability to create strong biofilm reported 93.33 %, 86.66 %, 86.66 % and 66.66 %, respectively.

**Conclusions:**

The findings indicated the importance of virulence genes in serogroups producing uropathogenic *E.coli* biofilm. It is recommended that strains producing biofilm before antibiotic use should be studied.

## Background

Urinary tract infections (UTI) are common causes of morbidity and sometimes lead to significant mortality. *Escherichia coli* (*E. coli*) was the most common etiological agent of UTI, accounting for nearly 80 % of community-acquired and 50 % of hospital-acquired infections [1] and tends to form microcolonies in mucosa lining of urinary bladder known as biofilm. These biofilms make the organism to resist the host immune response, more virulent and lead to the evolution of antibacterial drug resistance by enclosing them in an extracellular biochemical matrix [2]. Biofilms have a role in up to 60 % of human infections and they are very difficult to be eradicated with antimicrobial therapy. In vitro susceptibility tests have shown considerable increase in resistance of biofilm cells to killing [3]. Detection of biofilm-producer strains is therefore relevant for the design of adequate control measures for Uropathogenic *E. coli* (UPEC) infections.

The *Escherichia coli* strains are normally identified by serological typing of their H (flagellar), O (lipopolysaccharide) and in some cases, K (capsular) surface antigens. Overall, 184 O-serogroups are described for *E. coli* [4]. The O-serogroups of UPEC strains are related to certain virulence factor profile of each strain. Previous studies reported that O1, O2, O4, O6, O7, O8, O15, O16, O18, O21, O22, O25, O75 and O83 serogroups are preferentially associated with UPEC strains [5–13]. Some of the most important virulence genes of UPEC strains which are associated with severe UTIs are P fimbriae (*pap*), type 1 fimbriae, afimbrial adhesin I (*afaI*), hemolysin (*hly*), cytotoxic necrotizing factor 1 (*cnf 1*), aerobactin (*aer*), S fimbriae (*sfa*), adhesins and fimbriae [14, 15]; however, other virulence genes such as *kpsMT, ompT, usp, iroN, iha, set 1, astA*, group II capsule synthesis; *sfa/foc,* S and F1C fimbriae; *iutA, traT*, serum resistance; and *fimH*, are known to be involved in pathogenicity of this organism [16–18]. These Virulence factors help the organisms to colonize host surfaces, avoid and/or subvert host defense mechanisms, injure and/or invade host cells and tissues, and incite a noxious inflammatory response, thereby leading to clinical disease [19]. Uropathogenic *E. coli* strains more frequently produce *Pap* and *Prs* fimbrial adhesins. P fimbriae are important for colonization and persistence of *E. coli* in the colon and possibly facilitate spread to the urinary tract [20, 21]. The ability to constitute biofilm plays an important role in virulence of the bacteria, in addition to it; various genes encode urinary virulence factors.

In addition, several studies showed that antibiotic resistance in UPEC is increasing nowadays [22, 23]. Several studies have reported increasing trends in resistance against trimethoprim-sulfamethoxazole (TMP -SMZ) [24, 25] fluoroquinolones and other antibiotics, including ciprofloxacin [26, 27]. To reduce the rate of morbidity, an early treatment of UTIs is mandatory, which relays on empirical therapies. However, to initiate an effective empirical treatment, several factors must be taken into consideration, including geographical location, age and sex of the patient, and local antimicrobial resistance profiles of the pathogens.

The identification of bacterial resistance genes seems to be essential to reduce the treatment costs. Using phylogenetic grouping as defined by multilocus enzyme electrophoresis and muliplex polymerase chain reaction (PCR) assays, Johnson *et al*. reported detailed analyses about phylogenetic background and virulence attributes of uropathogenic *E. coli* strains isolated from urosepsis and cystitis [28]. To our knowledge, there is scarcity of data showing the common uropathogenic *E. coli* serogroups in causing urinary tract infections, antibiotic resistance pattern of strains and frequency distribution of types of virulence genes in isolations having the ability to constitute biofilm in Iran. Therefore, in this study, we determined to track the common uropathogenic *E. coli* serogroups, antimicrobial susceptibility patterns and the virulence gene distribution of UPEC strains having the ability to constitute biofilm isolated from patients with UTIs in Iran.

## Methods

### Bacterial strains and detection of uropathogenic *E. coli* serogroups and virulence genes

In the present study, a total of 130 *E. coli* strains isolates were isolated and collected from urine specimens of patients with UTI who that had been referred to the medical laboratory. The strains were isolated from pure cultures and identified and also confirmed biochemically and using molecular techniques in the laboratory.

The colonies were confirmed using Polymerase Chain Reaction (PCR) based on the detection of 16S rRNA gene region of *E. coli* described by Sabat et al., (2000) [29]. In addition, all isolates were serogrouped using PCR assays. Table 1 showed the primers used for detection of UPEC serogroups and the PCR conditions [30]. The oligonucleotide sequences and Multiplex Polymerase Chain Reaction conditions of the specific primers were used to amplify the *pap, fimH, sfa* and *afa* genes producing biofilm in uropathogenic *E. coli* are shown in Table 2 [30]. The amplified products were visualized by ethidium bromide staining after gel electrophoresis of 10 μL of the final reaction mixture in 1.5 % agarose.

**Table 1:** The oligonucleotide primers and the Multiplex PCR programs used for amplification of O-serogroups genes of *E. coli* isolates

Serotypes	Gene	Primer name	Primer Sequence (5'-3)	Size of product (bp)	PCR programs	M-PCR Volume (50 μL)
O1	*Wzx*	wl-14632	GTGAGCAAAAGTGAAATAAGGAACG	1098	1 cycle: 95 °^C^ ------------ 5 min. 30 cycle: 95 °^C^ ------------ 30 s 62 °^C^ ------------ 60 s 72 °^C^ ------------ 60 s 1 cycle: 72 °^C^ ------------ 5 min	5 μL PCR buffer 10X 2.5 mM Mgcl_2_ 300 μM dNTP (Fermentas) 0.4 μM of each primers F & R 2 U Taq DNA polymerase (Fermentas) 3 μL DNA template
		wl-14633	CGCTGATACGAATACCATCCTAC			
O6	*Wzy*	wl-14646	GGATGACGATGTGATTTTGGCTAAC	783		
		wl-14647	TCTGGGTTTGCTGTGTATGAGGC			
O7	*Wzx*	wl-14648	CTATCAAAATACCTCTGCTGGAATC	610		
		wl-14649	TGGCTTCGAGATTAAACCTATTCCT			
O8	*orf469*	wl-14652	CCAGAGGCATAATCAGAAATAACAG	448		
		wl-14653	GCAGAGTTAGTCAACAAAAGGTCAG			
O16	*Wzx*	wl-14654	GGTTTCAATCTCACAGCAACTCAG	302		
		wl-14655	GTTAGAGGGATAATAGCCAAGCGG			
O21	*Wzx*	wl-14676	CTGCTGATGTCGCTATTATTGCTG	209		
		wl-14677	TGAAAAAAAGGGAAACAGAAGAGCC			
O75	*Wzy*	wl-17413	GAGATATACATGGGGAGGTAGGCT	511		
		wl-17414	ACCCGATAATCATATTCTTCCCAAC			
O2	*Wzy*	wl-14636	AGTGAGTTACTTTTTAGCGATGGAC	770	1 cycle: 95 °^C^ ------------ 5 min. 30 cycle: 94 °^C^ ------------ 60 s 58 °^C^ ------------ 60 s	5 μL PCR buffer 10X 2.5 mM Mgcl_2_ 300 μM dNTP (Fermentas) 0.4 μM of each primers F & R 2 U Taq DNA polymerase (Fermentas)
		wl-14637	AGTTTAGTATGCCCCTGACTTTGAA			
O4	*Wzx*	wl-14642	TTGTTGCGATAATGTGCATGTTCC	664		
		wl-14643	AATAATTTGCTATACCCACACCCTC			
O15	*Wzy*	wl-14672	TCTTGTTAGAGTCATTGGTGTATCG	183		
		wl-14673	ATAAAACGAGCAAGCACCACACC			
O18	*Wzx*	wl-14656	GTTCGGTGGTTGGATTACAGTTAG	551		
		wl-14657	CTACTATCATCCTCACTGACCACG			
O22	*Wzx*	wl-14660	TTCATTGTCGCCACTACTTTCCG	468		
		wl-14661	GAAACAGCCCATGACATTACTACG			
O25	*Wzy*	wl-14666	AGAGATCCGTCTTTTATTTGTTCGC	230		
		wl-14667	GTTCTGGATACCTAACGCAATACCC			
O83	*Wzx*	wl-14668	GTACACCAGGCAAACCTCGAAAG	362		
		wl-14669	TTCTGTAAGCTAATGAATAGGCACC

**Table 2:** The oligonucleotide primers and the Multiplex PCR programs used for amplification of virulence genes of *E. coli* isolates

Gene	Primer name	Primer sequence (5'-3)	Size of product (bp)	PCR program	M-PCR volume (50 μL)
*pap*	*pap3* *pap4*	GCAACAGCAACGCTGGTTGCATCAT AGAGAGAGCCACTCTTATACGGACA	336	1 cycle: 94 °^C^ ------------ 1 min. 30 cycle: 94 °^C^ ------------ 60 s 63 °^C^ ------------ 30 s 72 °^C^ ------------ 90 s 1 cycle: 72 °^C^ ------------ 5 min	5 μL PCR buffer 10X 1.25 mM Mgcl_2_ 150 μM dNTP (Fermentas) 1 μM of each primers F & R 1.2 U Taq DNA polymerase (Fermentas) 3 μL DNA template
*Sfa*	*sfa1* *sfa2*	CTCCGGAGAACTGGGTGCATCTTAC CGGAGGAGTAATTACAAACCTGGCA	410		
*Afa*	*afa1* *afa2*	GCTGGGCAGCAAACTGATAACTCTC CATCAAGCTGTTTGTTCGTCCGCCG	750		
*fimH*	*FimH1* *FimH2*	GAGAAGAGGTTTGATTTAACTTATTG AGAGCCGCTGTAGAACTGAGG	559	1 cycle: 94 °^C^ ------------ 3 min. 40 cycle: 94 °^C^ ------------ 60 s 58 °^C^ ------------ 70 s 72 °^C^ ------------ 70 s 1 cycle: 72 °^C^ ------------ 6 min	5 μL PCR buffer 10X 2 mM Mgcl_2_ 200 μM dNTP (Fermentas) 0.4 μM of each primers F & R 1 U Taq DNA polymerase (Fermentas) 3 μL DNA template

### Detection of biofilm formation and antimicrobial susceptibility testing

All *E. coli* strains were included in the study and were analyzed for the production of biofilm and antimicrobial susceptibility pattern. Biofilm production in bacterial cultures was determined by Congo-red Agar method (CRA) as described previously by Solati et al. [31]. Congo-red was prepared as the aqueous solution, autoclaved, and then added when the agar cooled to 55 °C. Plates were inoculated and incubated for 24 hours at 37 °C. The positive isolate was indicated by black and dry crystalline colonies. Weak biofilm producers usually remained pink with the darkness at the center of colonies. Intermediate results were exhibited by the darkness of the colonies with the absence of a dry crystalline colonies.

Antimicrobial susceptibility testing was done by the Kirby–Bauer disc diffusion method using Mueller–Hinton agar (HiMedia Laboratories, Mumbai, India) according to the Clinical Laboratory Standards Institute (CLSI) guidelines [32] as has been previously described [30].

The antimicrobial agents tested and their corresponding concentrations were ampicillin (AM), tetracycline (TE), nalidixic acid (NA), co-trimoxazole (SXT), cephalothin (CF), ciprofloxacin (CP), norfloxacin (NOR), ceftriaxone (CRO), amikacin (AN), imipenem (IMP), gentamicin (GM) and nitrofurantoin (FM).

### Statistical analysis

SPSS version 17.0 statistical software package was used for statistical analysis. Chi-square test was applied. *P*-value < 0.05 was considered statistically significant

## Results

Among 130 *E.coli* isolates, 80 (61.53 %) were able to make biofilm. Among 80 *E. coli* strains subjected to biofilm production, 15 (18.75 %) strains showed highly positive with very black colonies color in Congo Red Agar (CRA), 20 strains (25 %) showed moderate positive with black colonies color in CRA, 45 strains (56.25 %) showed weakly positive with grey colonies color in CRA. Dry crystalline and black colonies at the Congo-red Agar culture, were considered as strong biofilm producers; isolates did not show dry crystalline black colonies were identified as moderately biofilm producers and non-biofilm producers showed pink or yellow colonies.

Antibiotic susceptibility pattern was studied for all *E. coli* isolates. The multi-drug resistant pattern of the biofilm producing and non producing UPEC *E. coli* is shown in Table 3. All the biofilm forming strains showed maximum resistance to ampicillin (87.5 %), followed by tetracycline (75 %), nalidixic Acid (72.5 %) and co-trimoxazole (71.25 %). Both biofilm producer and non- biofilm producer were highly resistant to ampicillin, followed by tetracycline and nalidixic acid. 93.75 % and 98 % sensitive was noticed for biofilm and non-biofilm producer against nitrofurantoin, respectively.

**Table 3:** Antibiotic resistance pattern of the biofilm producing and non producing Uropathogenic *E. coli*

Antibiotic	Biofilm producer (*N* = 80)		Non biofilm producer (*N* = 50)	
	Resistance	Sensitive	Resistance	Sensitive
Ampicillin (AM)	70 (87.5 %)	10 (12.5 %)	40 (80 %)	10 (20 %)
Tetracycline (TE)	60 (75 %)	20 (25 %)	35 (70 %)	15 (30 %)
Nalidixic Acid (NA)	58 (72.5 %)	22 (27.5 %)	34 (68 %)	16 (32 %)
Co-Trimoxazole (SXT)	57 (71.25 %)	23 (28.75 %)	33 (66 %)	17 (34 %)
Cephalothin (CF)	45 (56.25 %)	35 (43.75 %)	28 (56 %)	22 (44 %)
Ciprofloxacin (CP)	45 (56.25 %)	35 (43.75 %)	27 (54 %)	23 (46 %)
Norfloxacin (NOR)	43 (53.75 %)	37 (46.25 %)	26 (52 %)	24 (48 %)
Ceftriaxone (CRO)	33 (41.25 %)	47 (58.75 %)	20 (40 %)	30 (60 %)
Amikacin (AN)	31 (38.75 %)	49 (61.25 %)	18 (36 %)	32 (64 %)
Imipenem (IMP)	25 (31.25 %)	55 (68.75 %)	15 (30 %)	35 (70 %)
Gentamicin (GM)	15 (18.75 %)	65 (81.25 %)	9 (18 %)	41 (82 %)
Nitrofurantoin (FM)	5 (6.25 %)	75 (93.75 %)	1 (2 %)	49 (98 %)

Our results revealed high distribution of UPEC serogroups isolated from patients with urinary tract infection. Totally, O25 (26.66 %), O15 (20.0 %) and O16 (13.33 %) had the highest biofilm producing serogroups while O2, O4, O6, O8, O21 and O22 had the lowest biofilm producing serogroups which showed (6.66 %) among Uropathogenic *E. coli* isolates detected (Table 4).

**Table 4:** Prevalence of serogroups Uropathogenic *E. coli*

Number of positive samples	Prevalence of serogroups (%)													
	O1	O2	O4	O6	O7	O8	O15	O16	O18	O21	O22	O25	O75	O83
High Biofilm Production *E. coli* (*N* = 15)	-	1 (6.66 %)	1 (6.66 %)	1 (6.66 %)	-	1 (6.66 %)	3 (20 %)	2 (13.33 %)	-	1 (6.66 %)	1 (6.66 %)	4 (26.66 %)	-	-
Moderate Biofilm Production *E. coli* (*N* = 20)	1 (5 %)	1 (5 %)	1 (5 %)	1 (5 %)	1 (5 %)	1 (5 %)	3 (15 %)	2 (10 %)	1 (5 %)	1 (5 %)	1 (5 %)	5 (25 %)	1	-
Weak Biofilm Production *E. coli* (*N* = 45)	1 (2.22 %)	1 (2.22 %)	2 (4.44 %)	6 (13.33 %)	1 (2.22 %)	1 (2.22 %)	10 (22.22 %)	5 (11.11 %)	1 (2.22 %)	2 (4.44 %)	1 (2.2 %)	13 (28.88 %)	1 (2.22 %)	-
No biofilm *E. coli* (*N* = 50)	1 (2 %)	1 (2 %)	3 (6 %)	8 (16 %)	2 (4 %)	1 (2 %)	11 (22 %)	1 (2 %)	1 (2 %)	4 (8 %)	1 (2 %)	14 (28 %)	1 (2 %)	1 (2 %)

In the present study the prevalence of *fimH*, *pap*, *sfa* and *afa* genes in Uropathogenic *E. coli* was determined and the result showed that among high biofilm producer Uropathogenic *E. coli* isolates *fimH* gene was the highest prevalence and *afa* gene was the lowest prevalence virulence gene (Table 5). Biofilm production was significantly associated with *fimH*, *pap*, *afa* and *sfa* virulence genes (*P* < 0.05).

**Table 5:** Prevalence of *fimH*, *pap*, *sfa* and *afa* genes in Uropathogenic *E. coli*

Virulence gene	UPEC *E. coli*				
	High biofilm producer 15	Moderate biofilm producer 20	Weak biofilm producer 45	Non biofilm producer 50	*P* value
*fimH*	14 (93.33 %)	18 (90 %)	35 (77.77 %)	30 (60 %)	0.031
*pap*	13 (86.66 %)	16 (80 %)	30 (71.42 %)	28 (56 %)	0.001
*sfa*	13 (86.66 %)	10 (50 %)	20 (44.44 %)	20 (40 %)	0.033
*afa*	10 (66.66 %)	7 (35 %)	15 (33.33 %)	10 (20 %)	0.035

## Discussion

Urinary tract infections are among the most common bacterial diseases worldwide which involve (infects) about 250 million people in developing countries annually [33, 34]. Uropathogenic *E. coli* alone account for 70-90 % of the UTI infections [35, 36] and their susceptibility patterns against different antibiotics vary in different geographical regions, eventually leading to empirical therapy which is based on the local susceptibility profiles. Bacterial biofilm are often associated with long-term persistence of organism in various environments. Bacteria in biofilm display dramatically increased resistance to antibiotics [37].

Among 80 *E. coli* isolates subjected to biofilm production, 15 (18.75 %) isolates showed highly positive, 20 isolates (25 %) showed moderate positive, 45 isolates (56.25 %) showed weakly positive in Congo Red Agar method (CRA). Antibiotic susceptibility pattern was studied for all *E. coli* isolates.

The biofilm forming isolates showed maximum resistance to Ampicillin (87.5 %), followed by Tetracycline (75 %), Nalidixic Acid (72.5 %) and Co-Trimoxazole (71.25 %). Both biofilm producer and non- biofilm producer were highly resistant to Ampicillin, followed by Tetracycline and Nalidixic Acid. 93.75 % and 98 % sensitivity was noticed for biofilm and non-biofilm producer against Nitrofurantoin, respectively. The findings of the current investigations are in agreement with the reports of Reisner et al. [38] ; Ong et al. [39]; Ulett et al. [40] and Ulett et al. [41] in which a greater variation was observed against the uropathogenic *E. coli* forming biofilms under different conditions. Another finding of this study is that strong biofilm producers were less susceptible to antimicrobial agents than the non-biofilm producer. This result may agree with the previous studies showing that the sessile bacterial cells seems to exhibit higher resistance than the planktonic cells [42–48], so the findings of the current investigation indicated that resistance mechanisms are associated with the formation of biofilm among uropathogenic *E. coli.*

Similarly, the increasing prevalence of multi-drug resistance (MDR) has been reported by other workers showed, of the 100 (60.2 %) *E. coli* strains, 72 strains displayed a biofilm positive phenotype under the optimized conditions in the Congo Red agar medium and the strains were classified as highly positive (17, 23.6 %), moderate positive (19, 26.3 %) and weakly positive (36, 50.0 %). The rates of antibiotic resistance of biofilm producing *E. coli* were found to be 100 % for chloramphenicol and amoxyclav (amoxicillin and clavulanic acid), 86 % for gentamicin and cefotaxime, 84 % for ceftazidime, 83 % for cotrimoxazole and piperacillin/tazobactam, 75 % for tetracycline and 70 % for amikacin [49]. This could be due to dissemination of MDR strains in hospital settings and the different combination of antibiotics resulted in varying degree of resistance among the biofilm producing uropathogenic *E. coli*.

In addition, 56.25 % of biofilm-producing UPEC isolates showed resistance to Cephalothin. Similar findings had been previously observed in South East Asian region [50–52]. Reported resistance rate against these drugs was comparatively lower in previous study in Iran (19.6 %) [53] and in Bangladesh, it was 32 % [54]. However, observed higher percentages of resistances against Cephalothin drugs indicated that they could render their efficacies as therapeutic agents, particularly in Iranian population. In general, our results suggest that transformation of UTI-associated *E. coli* with plasmids carrying different antibiotic resistance gene had a significant impact on biofilm formation and that these effects were both strain dependent and varied between different antibiotics.

Our results revealed high distribution of UPEC serogroups isolated from patients with urinary tract infection. Totally, O25 (26.66 %), O15 (20.0 %) and O16 (13.33 %) had the highest biofilm producing serogroups while O2, O4, O6, O8, O21 and O22 had the lowest biofilm producing serogroups which showed (6.66 %) among Uropathogenic *E. coli* isolates detected (Table 4). In the present study the prevalence of *fimH*, *pap*, *sfa* and *afa* genes in Uropathogenic *E. coli* was determined and the result showed that among High biofilm producer Uropathogenic *E. coli* isolates *fimH* gene was the highest prevalence and *afa* gene was the lowest prevalence virulence gene (Table 5). Biofilm production was significantly associated with *fimH*, *pap*, *afa* and *sfa* virulence genes (*P* < 0.05). Manuela et al. reported that Biofilm production was significantly associated with fluoroquinolone resistance at all incubation time points and was independent of the media used (*P* < 0.05). Biofilm production was not associated with *cnf1, hly, pap* and *sfa* genes (*P* > 0.05), but was significantly associated with *afa, aer* and the β-lactamase genes (*P* < 0.05) [55].

## Conclusions

Urinary tract infections are one of the common infections which are encountered in the clinical practice. This study reveals the prevalence and antimicrobial susceptibility pattern of biofilm and non-biofilm producing uropathogenic *E. coli* strains. Biofilm formation is closely related with the resistance of *E. coli* towards the antimicrobial drugs and also it increases the chronicity of urinary tract infection. In general, the current study demonstrated a high tendency among the clinical isolates of *E. coli* to form biofilm. The present study has also shown the production of various virulent factors and developing drug resistance in UPEC. Antibiotic resistance may provide a substantial advantage to the survival of the pathogen. The drug resistance among UPEC is on rise therefore the selection of appropriate antibiotics (after antibiotic susceptibility testing) is must for proper treatment of patients and to avoid emergence of drug resistance. Therefore, the knowledge of virulence factors of *E. coli* and their antibiotic susceptibility pattern will help in better understanding of the organism and in the treatment of UTI.

## Abbreviations

UPEC: Uropathogenic E. coli
UTIs: Urinary Tract Infections
E. coli: Escherichia coli
aer: Aerobactin
pap: P fimbriae
afa I: Type 1 fimbriae, afimbrial adhesin
hly: Hemolysin
cnf 1: Cytotoxic necrotizing factor 1
sfa: S fimbriae
PCR: Polymerase chain reaction
CRA: Congo red agar
CLSI: Clinical and Laboratory Standards Institute
MHA: Mueller Hinton agar
AM: Ampicillin
TE: Tetracycline
NA: Nalidixic acid
SXT: Co-trimoxazole
CF: Cephalothin
CP: Ciprofloxacin
NOR: Norfloxacin
CRO: Ceftriaxone
AN: Amikacin
IMP: Imipenem
GM: Gentamicin
FM: Nitrofurantoin
SPSS: Statistical package for the social sciences
